# Ferulic Acid Alleviates Inflammation and Promotes Osteogenic Differentiation in Periodontitis by Inhibiting NF‐*κ*B Pathway

**DOI:** 10.1155/sci/1891956

**Published:** 2025-12-17

**Authors:** Qiao Wang, Bo Feng, Yongzhi Gao

**Affiliations:** ^1^ Department of Stomatology, The Second Affiliated Hospital of Qiqihar Medical University, Qiqihar, 161006, China; ^2^ Department of Stomatology, Qiqihar Wuguan Hospital, Qiqihar, 161006, China; ^3^ Department of Stomatology, The First Affiliated Hospital of Qiqihar Medical University, Qiqihar, 161006, China

**Keywords:** ferulic acid, inflammation, NF-*κ*B pathway, osteogenic differentiation, periodontitis

## Abstract

**Background:**

Periodontitis refers to a chronic inflammatory illness that induces the destruction of periodontal tissues and can be driven by bacterial lipopolysaccharide (LPS) of pathogens. This study investigated the anti‐inflammatory potential and underlying mechanisms of ferulic acid (FA) in periodontitis.

**Method:**

An in vitro periodontitis model was established by treating human periodontal ligament stem cells (hPDLSCs) with 10 µg/mL LPS for 24 h. The experimental groups included a control group, an LPS‐treated group, and an LPS + FA cotreatment group. In addition, phorbol 12‐myristate 13‐acetate (PMA) treatment was used for nuclear factor *κ*B (NF‐*κ*B) pathway activation. Cell proliferation was evaluated using the Cell Counting Kit‐8 (CCK‐8) test, and osteogenic differentiation was measured by alkaline phosphatase (ALP) and alizarin red S (ARS) staining. Apoptosis was detected with flow cytometry utilizing Annexin V‐APC/PI double staining. Protein expressions were measured by Western blot. Inflammatory cytokine secretion was measured via enzyme‐linked immunosorbent assay (ELISA) kits.

**Result:**

This study uncovered that FA alleviates LPS‐induced inflammatory responses in hPDLSCs, promoting cell proliferation and osteogenic differentiation. FA inhibits NF‐*κ*B pathway activation, reduces proinflammatory cytokines (tumor necrosis factor‐alpha [TNF‐*α*], interleukin [IL]‐1*β*, IL‐6), increases anti‐inflammatory cytokine IL‐10, and upregulates osteogenic markers (runt‐related transcription factor 2 [Runx2], type I collagen [COL1], osteopontin [OPN], osteocalcin [OCN]). However, the protective effects of FA are reversed by the NF‐*κ*B activator PMA, indicating that its therapeutic efficacy primarily depends on NF‐*κ*B signaling regulation.

**Conclusion:**

This study evaluated FA’s effects on inflammation and osteogenic function in LPS‐induced hPDLSCs, revealing its potential to alleviate periodontitis via NF‐*κ*B pathway inhibition and identifying a possible therapeutic target for periodontal disease.

## 1. Introduction

Periodontitis is a chronic inflammatory disease linked to dental plaque accumulation, characterized by progressive loss of tooth‐supporting structures, encompassing the alveolar bone and periodontal ligament [1, 2]. Clinically, the disease presents with gingival inflammation, loss of periodontal attachment and alveolar bone, tooth mobility and loss, pathological tooth migration, deep probing depths, and bleeding upon probing [3, 4], seriously affecting the chewing performance. Periodontitis pathogenesis is driven by the interplay among microbial pathogens, host immune responses, and environmental influences, such as food residue and smoking [5]. These periodontal pathogens (like *Tannerella forsythia*, *Treponema denticola* and *Porphyromonas gingivalis*) or their products (lipopolysaccharide [LPS] and virulence factors) stimulated the host macrophages and other inflammatory cells [6], which were activated to restrict bacterial dissemination through producing a series of proinflammatory cytokines (interleukin [IL]‐1*β*, tumor necrosis factor [TNF]‐*α*, prostaglandin E2 [PGE2]) [7] and also exhibited crucial destruction characteristic of the alveolar bone, periodontal ligament and connective tissue of periodontitis [8]. Current treatments mainly involve the use of scaling and root planning (SRP) and antimicrobials to lower bacterial load, with host modulators also used to curb chronic inflammation [9]. However, this treatment strategy may lead to issues such as drug resistance and microbial imbalance, and it has shown limited efficacy in promoting the functional regeneration of periodontal supporting tissues like alveolar bone [10]. Furthermore, alveolar bone loss mediated by osteoblast dysfunction remains a key irreversible aspect of periodontal disease [11]. Therefore, there is an urgent need for therapeutic approaches that suppress periodontitis inflammation while simultaneously promoting functional alveolar bone regeneration.

Ferulic acid (FA) is a type of phenolic acid in the cell wall of many plants, such as *Ferula asafetida* L., *Ligusticum chuanxiong* Hort., *Cimicifuga foetida* L., and *Angelica sinensis* [12], with strong antiviral and antioxidant, anti‐inflammatory, antithrombosis, antitumor, and neuroprotective activity [13]. Based on these promising biological properties, FA has gained attention for its potential application in various therapeutic fields. For example, a FA‐loaded hydrogel was developed to combat ROS‐induced oxidative stress during wound healing, effectively enhancing antioxidant enzyme activity (SOD, GPx), reducing ROS and MDA levels, and promoting tissue regeneration and collagen synthesis [14]. In a clinical trial of 20 periodontal patients, the results showed that FA hydrogel had low hemolytic activity and minimal toxicity to fibroblasts, and significantly reduced the pocket depth (PD), clinical attachment loss (CAL), plaque index, and gingival index (GI), indicating good biocompatibility and therapeutic potential [15]. Another study demonstrated that pycnogenol significantly lowered inflammatory markers matrix metalloproteinase‐8 (MMP‐8) and IL‐6 in patients with gingival inflammation following plaque removal, accompanied by elevated salivary FA levels, highlighting FA as a key bioactive metabolite potentially responsible for the anti‐inflammatory effects and improved periodontal outcomes [16].

Proinflammatory cytokines stimulated osteoblasts and T helper cells to express receptor activator of nuclear factor *κ*B ligand (RANKL), which then binds to the receptor of RANK on osteoclast precursors, accelerating their differentiation into mature osteoclasts. These mature osteoclasts then drive alveolar bone resorption [17]. Inhibiting the nuclear factor *κ*B (NF‐*κ*B) pathway activation can reduce inflammation, facilitate osteogenic differentiation of human periodontal ligament stem cells (hPDLSCs) [18, 19], and ultimately improve periodontitis [20]. Research has reported that FA shows anti‐inflammatory roles by suppressing the TLR4/NF‐*κ*B pathway, aiding nerve repair, and relieving sciatic neuropathy [21]. In rheumatoid arthritis, FA inhibited osteoclast differentiation and bone erosion through suppression of the RANKL‐dependent NF‐*κ*B pathway [22]. Additionally, FA promotes bone regeneration after radiation‐induced defects by preserving skeletal stem cell stemness [23] and enhances osteogenic differentiation of bone marrow mesenchymal stem cells (BMMSCs) in vitro through downregulating miR‐340 and activating *β*‐catenin [24]. However, the underlying mechanisms of FA in periodontitis remain largely unexplored. Therefore, this research investigates the anti‐inflammatory and bone‐protective roles of FA in periodontitis and elucidates its potential molecular mechanisms, offering a theoretical basis for its development as a novel treatment agent for periodontal disease.

## 2. Materials and Methods

### 2.1. Periodontitis Model Establishment and Proliferative Activity Assessment

hPDLSCs were purchased from the Pricella Biotechnology Co., Ltd (CP‐H234, Hubei, Wuhan, China), and the FA was obtained from the MedChemExpress company (HY‐N0060, Shanghai, China). The cells were cultured in complete growth medium consisting of *α*‐MEM (Gibco, USA) supplemented with 10% fetal bovine serum (FBS, Gibco, USA), 1% penicillin‐streptomycin solution (Beyotime, China), and 2 mM L‐glutamine [25]. Cells were maintained in a humidified incubator at 37°C with 5% CO_2_. The culture medium was refreshed every 2–3 days. All experiments were performed using cells between passages 3 and 5 to ensure optimal cell viability and phenotypic stability. Notably, short tandem repeat (STR) identification has been carried out on the cells, and the outcome of mycoplasma detection shows negativity.

In addition, the cells were pretreated with 30 µM FA for 24 h in complete culture medium, then received the 10 µg/mL ultrapure LPS from *Porphyromonas gingivalis* (InvivoGen, USA) stimulation for 24 h to reveal the impact of FA on periodontitis [23]. The experiments were split into 3 groups comprising the control, LPS, and LPS + FA groups. Cell Counting Kit‐8 (CCK‐8; C0037) kit from the Beyotime Biotechnology Co., Ltd (Shanghai, China) was used for cell proliferation assay. 4 × 10^3^ cells/well were injected in the 96‐well plate with complete medium for 24, 48, and 72 h, and 10 μL of CCK‐8 was added for 1‐h incubation, and the absorbance at 450 nm was detected with a microplate reader. NF‐*κ*B pathway activator of phorbol 12‐myristate 13‐acetate (PMA; HY‐18739) from MedChemExpress was employed to induce NF‐*κ*B pathway activation and study its downstream effects on inflammation, cell survival, and immune responses. The hPDLSCs were pretreated with 100 nM of PMA for 30 min [26], followed by treatment with 30 μM FA and 10 *μ*g/mL LPS as the LPS + FA + PMA group.

### 2.2. Alkaline Phosphatase (ALP) and Alizarin Red S (ARS) Staining

ALP serves as an early marker of osteoblast differentiation and is crucial for osteogenesis. ALP staining can help assess the osteogenic differentiation of hPDLSCs in vitro. In addition, ALP staining is commonly combined with ARS staining, which identifies calcium deposition, to provide a comprehensive assessment of osteogenic differentiation [27]. We cultured the hPDLSCs from different treatment groups in osteogenic induction medium (CN417D‐250, Sigma–Aldrich, USA) for 14 days to induce osteogenic differentiation [28], with the medium refreshed every 2–3 days. And the ALP staining was conducted on day 7 by BCIP/NBT Alkaline Phosphatase Color Development Kit (C3206, Beyotime, Shanghai, China), cells were fixed with 4% paraformaldehyde for 10–15 min at room temperature and then washed with PBS. ALP staining solution (BCIP/NBT working solution) was applied, and samples were stored in the dark for 15–30 min. After rinsing with PBS or distilled water, stained cells were visualized by a microscope. ARS staining on day 14 was used to evaluate osteogenic differentiation. Cells were fixed with the fixative solution for 20 min, then washed with PBS three times. An appropriate amount of ARS staining solution (C0148S, Beyotime) was added to evenly cover the cells, and staining was performed at room temperature for 30 min. Superfluous dye was eliminated, and representative pictures were taken with a microscope.

### 2.3. Flow Cytometry

The cell apoptosis was measured by flow cytometry. First, hPDLSCs are collected from culture dishes, centrifuged to remove the supernatant, and resuspended in PBS for washing to eliminate residual medium. Cells (3 × 10^5^) were harvested and resuspended in 100 μL of 1x binding buffer. For staining, 5 μL Annexin V‐APC and 5 μL PI solution was added in dark for 10 min incubation using Annexin V‐APC/PI Apoptosis Detection Kit (A214‐01, Vazyme, Nanjing, China), Annexin V binds specifically to phosphatidylserine (PS), indicating early apoptosis, while PI enters only late apoptotic or necrotic cells, allowing differentiation between live, early apoptotic, late apoptotic, and necrotic cells [29, 30]. Finally, labeled samples are analyzed using a flow cytometer (BD Biosciences, Franklin Lakes, NJ, USA), and subsequent data analysis was conducted using FlowJo software (BD Biosciences, v.9.9.6).

### 2.4. Western Blot for the Osteogenic Related Proteins

Western blot was employed for evaluating the expression of osteogenic markers like runt‐related transcription factor 2 (Runx2), type I collagen (COL1), osteopontin (OPN), and osteocalcin (OCN) during the osteogenic differentiation of hPDLSCs [19, 25]. The process involves lysing cells to extract total protein, quantifying protein concentration with the BCA assay Kit (P0009, Beyotime), separating proteins via SDS–PAGE, and transferring them onto a PVDF or nitrocellulose membrane. After blocking with 5% skim milk, the membrane is incubated with primary antibodies including Runx2 (ab236639, 1:1000, Abcam), COL1 (ab275746, 1:1000, Abcam), OPN (ab75285, 1:1000, Abcam), OCN (ab133612, 1:1000, Abcam) overnight at 4°C, and the goat anti‐rabbit IgG secondary antibody conjugated to horseradish peroxidase was added for 1 h incubation at room temperature. In addition, the NF‐*κ*B pathway is imperative for immune and inflammatory responses, as well as cell growth and survival. Phosphorylation levels of NF‐*κ*B pathway‐related proteins, such as p‐p65 (ab76302, 1:1000, Abcam), p65 (ab32536, 1:1000, Abcam), p‐I*κ*B*α* (ab92700, 1:1000, Abcam), I*κ*B*α* (ab32518, 1:1000, Abcam), and GAPDH (ab8245, 1:1000, Abcam), were detected for the NF‐*κ*B pathway activation assessment [31]. Finally, chemiluminescence is used to detect the protein signals (E422‐01, Vazyme), which are analyzed by densitometry to determine relative expression levels.

### 2.5. Enzyme‐Linked Immunosorbent Assay (ELISA)

The ELISA kit was employed for the inflammatory factor detection to assess the underlying anti‐inflammatory effect of FA treatment in LPS‐induced hPDLSCs. The proinflammatory factor of TNF‐alpha (TNF‐*α*), IL‐1*β*, IL‐6, and the anti‐inflammatory factor of IL‐10 [26, 28, 32] were examined using the ELISA kit (H052‐1‐2 for TNF‐*α*, H002‐1‐2 for IL‐1*β*, H007‐1‐1 for IL‐6, H009‐1‐2 for IL‐10) from the Nanjing Jiancheng Bioengineering Institute (Nanjing, China). The ELISA process involves preparing samples (cell supernatant), coating microplate wells with capture antibodies overnight, and blocking to prevent nonspecific binding. Standards and samples were incubated, followed by washing and addition of biotinylated detection antibodies; cytokine concentrations are determined using the standard curve.

### 2.6. Statistical Analysis

Statistical analysis was conducted by the GraphPad Prism (version 9.0) software [33]. For comparisons of parameters across multiple groups under a single factor, one‐way analysis of variance (ANOVA) was employed, followed by Tukey’s post hoc test for multiple comparisons. Student’s *t*‐test was applied to analyze differences in experiments involving the NF‐*κ*B activator PMA. All data are presented as mean ± standard deviation (SD) from at least three independent experiments. A *p*‐value of less than 0.05 was considered statistically significant.

## 3. Results

### 3.1. FA Promotes the Proliferative Activity of hPDLSCs Under LPS Stimulation

First, we found that it promotes the proliferation of hPDLSCs in a dose‐dependent manner, with the optimal effect observed at a concentration of 30 μM (Figure S1). We detected the cell proliferation of hPDLSCs in different groups, revealing that the cell proliferation ability was markedly decreased in the LPS‐induced and LPS + FA treatment group in comparison to the control group, but the cell activity in the LPS + FA was notably higher than the LPS group (Figure 1A), suggesting that FA may play a protective role in periodontitis. To further investigate the protective effect of FA, we analyzed cell apoptosis by flow cytometry (Figure 1B,C). Quantitative analysis revealed that LPS induction dramatically increased the total apoptosis rate to 11.21%, with early apoptosis accounting for 8.94% and late apoptosis for 2.27%. In contrast, FA treatment significantly reduced the total apoptosis rate to 3.54%, predominantly by decreasing the proportion of early apoptotic cells from 8.94% to 3.52%. These findings demonstrate that FA effectively mitigates LPS‐induced apoptosis in hPDLSCs, particularly in the early stages of programmed cell death.

Figure 1The effect of ferulic acid on cell proliferation and apoptosis. (A) CCK‐8 for cell proliferation assessment. (B, C) Flow cytometry for detecting cell apoptosis. All procedures were performed in triplicate (*n* = 3) ( ^∗∗^ *p* < 0.01,  ^∗∗∗^ *p* < 0.001,  ^∗∗∗∗^ *p* < 0.0001).

**Figure:**
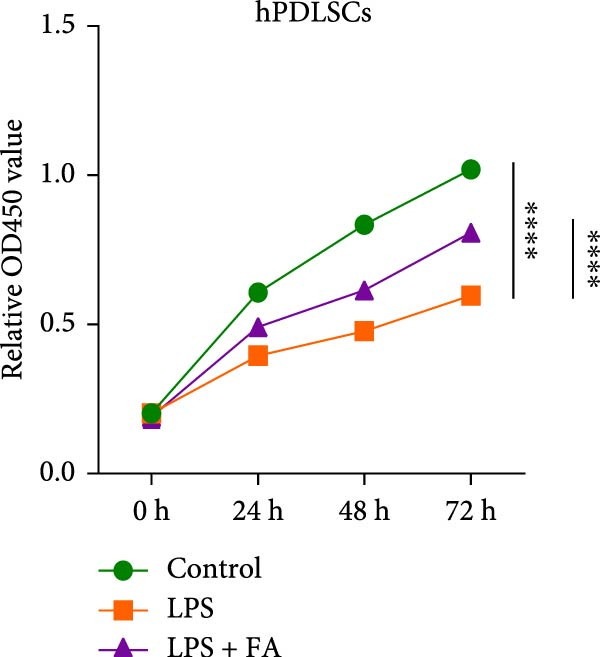
(A)

**Figure:**
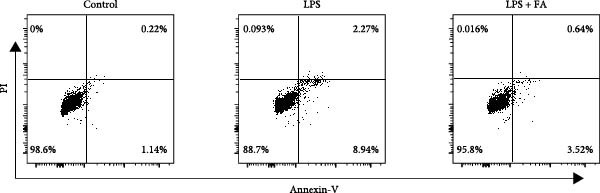
(B)

**Figure:**
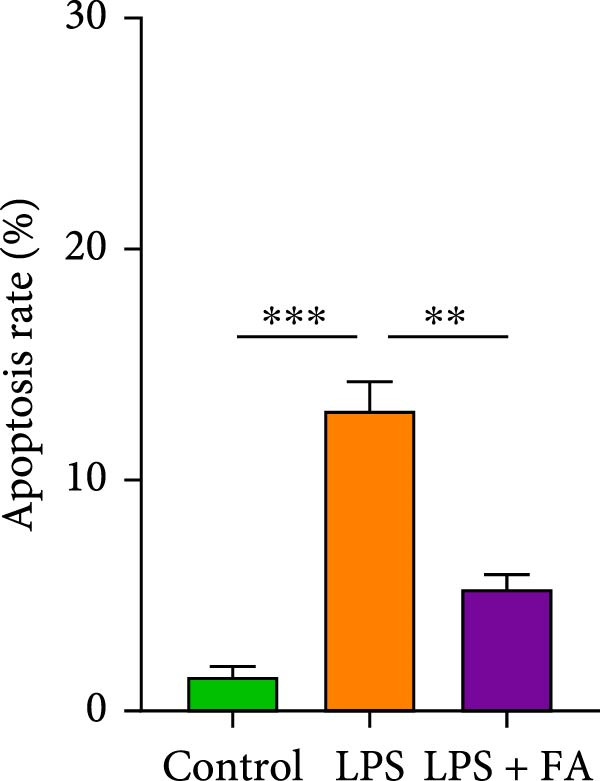
(C)

### 3.2. FA Reverses LPS‐Induced Inhibition of Osteogenic Differentiation in hPDLSCs

In periodontitis, inflammation activates molecular and cellular processes that boost osteoclast activity, causing alveolar bone loss. It also impairs osteoblast function, limiting new bone formation. ALP serves as an early marker of osteoblast differentiation and is elevated during osteogenic induction. Staining with substrates like BCIP/NBT produces a blue precipitate, enabling visualization of ALP activity under a microscope. After 7 and 14 days of osteoblast differentiation induction, ALP staining showed reduced ALP activity in the LPS‐treated group, but the FA treatment increased the osteogenic differentiation of hPDLSCs in the LPS + FA group (Figure 2A). ARS is a pH‐sensitive dye that binds Ca^2+^ to form insoluble red or orange‐red complexes. The ARS staining examined the calcium deposit level of hPDLSCs, and results revealed that the LPS induction inhibited the formation of calcium nodules with the decrease of Ca^2+^ insoluble complexes in hPDLSCs, while FA treatment promoted calcium nodules in hPDLSCs (Figure 2A). Further, we detected the osteoblast differentiation‐related protein expression in hPDLSCs; Western blot assay showed that the protein levels of Runx2, COL1, OPN, and OCN were markedly decreased in the LPS group, while FA pretreatment significantly reversed this reduction (Figure 2B).

Figure 2The impact of ferulic acid on osteoblast differentiation. (A) Alkaline phosphatase (ALP) and alizarin red S (ARS) staining for osteoblast differentiation. (B) Western blot for the osteoblast differentiation‐related protein expression ( ^∗^ *p* < 0.05,  ^∗∗^ *p* < 0.01,  ^∗∗∗^ *p* < 0.001,  ^∗∗∗∗^ *p* < 0.0001).

**Figure:**
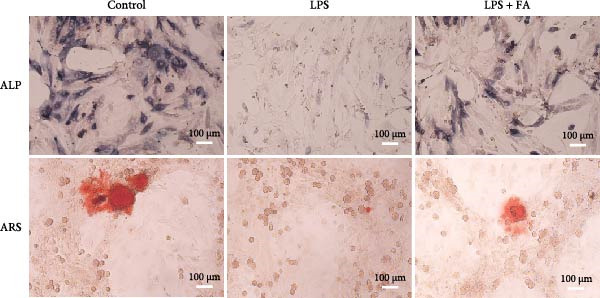
(A)

**Figure:**
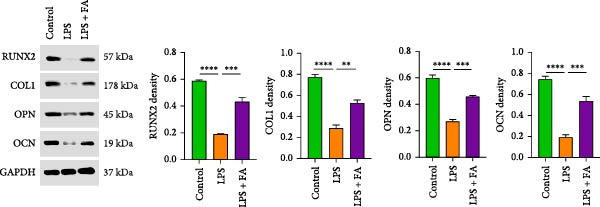
(B)

### 3.3. FA Reduces Proinflammatory Cytokine Production in hPDLSCs

Meanwhile, FA treatment also reduced TNF‐*α*, IL‐1*β*, and IL‐6 levels (proinflammatory factors), but enhanced IL‐10 level (anti‐inflammatory factor) (Figure 3A). The p‐p65 and p‐I*κ*B*α* levels in the NF‐*κ*B pathway for inflammatory response activation were increased after LPS induction, but their expressions were markedly decreased after FA treatment (Figure 3B), reflecting that FA treatment can alleviate inflammation in LPS‐stimulated hPDLSCs.

Figure 3The impact of ferulic acid on inflammatory factor production and NF‐*κ*B pathway activation. (A) Elisa for the TNF‐*α*, IL‐1*β*, IL‐6, and IL‐10 levels assessment. (B) Western blot for the phosphorylation of P65 and IкBa levels ( ^∗^ *p* < 0.05,  ^∗∗^ *p* < 0.01,  ^∗∗∗^ *p* < 0.001,  ^∗∗∗∗^ *p* < 0.0001).

**Figure:**
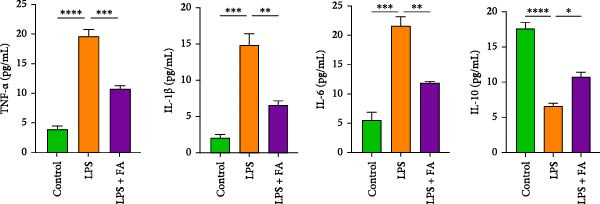
(A)

**Figure:**
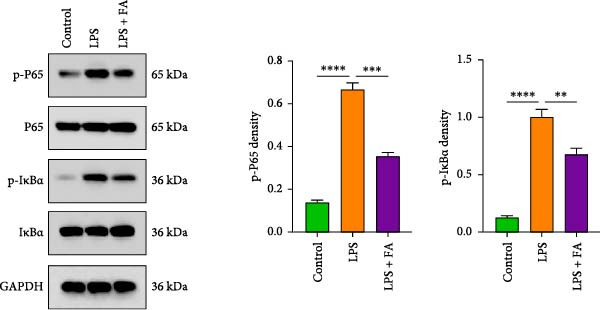
(B)

### 3.4. Inhibiting NF‐*κ*B Pathway Contributes to the Anti‐Inflammatory and Pro‐Osteogenic Effects of FA in LPS‐Stimulated hPDLSCs

For further determining the impact of FA treatment on the NF‐*κ*B pathway, we compared the inflammatory levels in the LPS + FA and the LPS + FA + PMA groups. Consistently, the TNF‐*α*, IL‐1*β*, and IL‐6 levels were notably enhanced, yet the IL‐10 level was markedly reduced in the LPS + FA + PMA group (Figure 4A), and the p‐p65 and p‐I*κ*B*α* levels were increased for the NF‐*κ*B pathway activation in the LPS + FA + PMA group (Figure 4B). These results implied that the LPS induced NF‐*κ*B pathway activation for inflammatory response in periodontitis, but FA treatment inhibited NF‐*κ*B pathway activation, alleviating the progression of periodontitis. CCK‐8 test and flow cytometry revealed the impaired cell proliferation and increased cell apoptosis in the LPS + FA + PMA group (Figure 5A,B), and the ALP and ARS staining damaged the osteoblast differentiation and calcium deposit level in the LPS + FA + PMA group (Figure 5C), with the decreased osteoblast‐related proteins (Runx2, COL1, OPN, and OCN) levels compared with the LPS + FA group (Figure 5D,E). Our findings suggest that FA alleviates the inflammatory response in LPS‐stimulated hPDLSCs through refraining the NF‐*κ*B pathway, and promotes their proliferation and osteogenic differentiation in periodontitis.

Figure 4The impact of ferulic acid on inflammatory factors after NF‐*κ*B pathway activation. (A) Elisa for the TNF‐*α*, IL‐1*β*, IL‐6, and IL‐10 levels assessment after NF‐*κ*B pathway activation. (B) Western blot for the phosphorylation of P65 and IкBa levels after NF‐*κ*B pathway activation ( ^∗^ *p* < 0.05,  ^∗∗^ *p* < 0.01).

**Figure:**
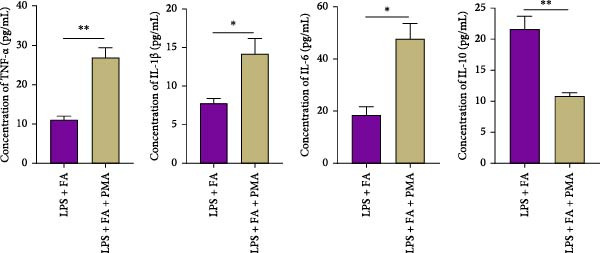
(A)

**Figure:**
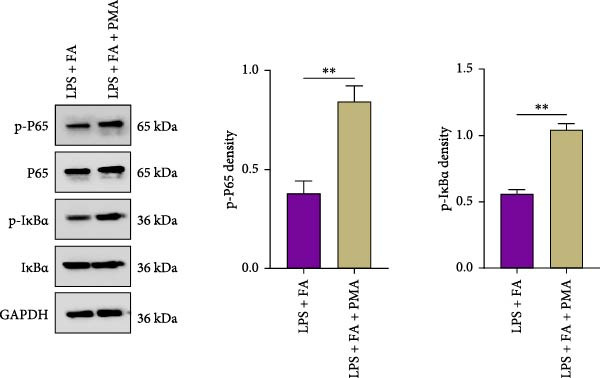
(B)

Figure 5The impact of ferulic acid on cell apoptosis and proliferation, and osteoblast differentiation after NF‐*κ*B pathway activation. (A) CCK‐8 for cell proliferation after NF‐*κ*B pathway activation. (B) Flow cytometry for detecting cell apoptosis after NF‐*κ*B pathway activation. (C) ALP and ARS staining for osteoblast differentiation after NF‐*κ*B pathway activation. (D, E) Western blot for the osteoblast differentiation‐related protein expression after NF‐*κ*B pathway activation ( ^∗∗^ *p* < 0.01,  ^∗∗∗^ *p* < 0.001,  ^∗∗∗∗^ *p* < 0.0001).

**Figure:**
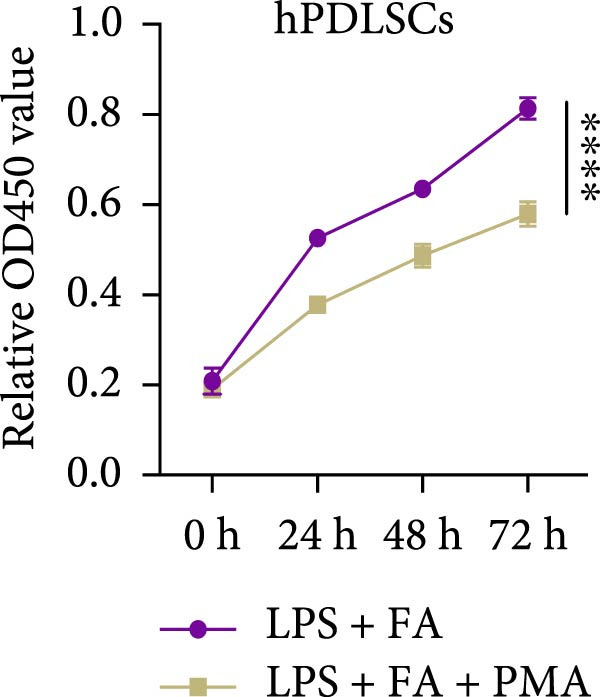
(A)

**Figure:**
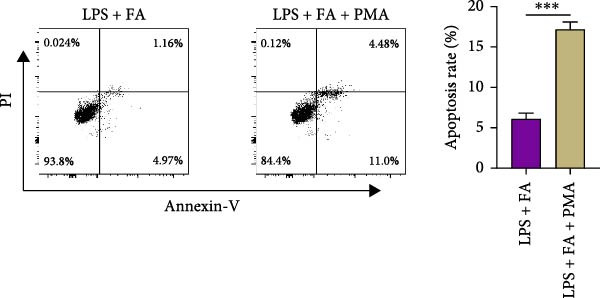
(B)

**Figure:**
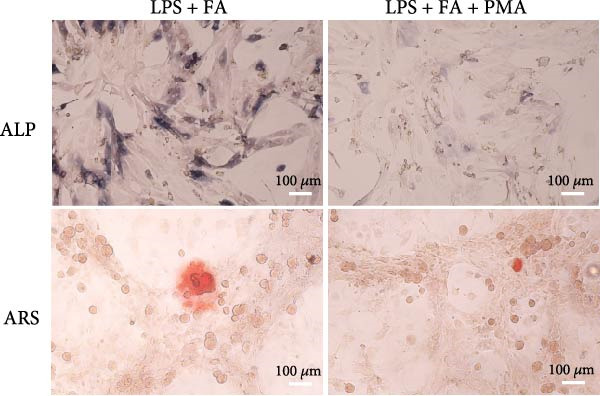
(C)

**Figure:**
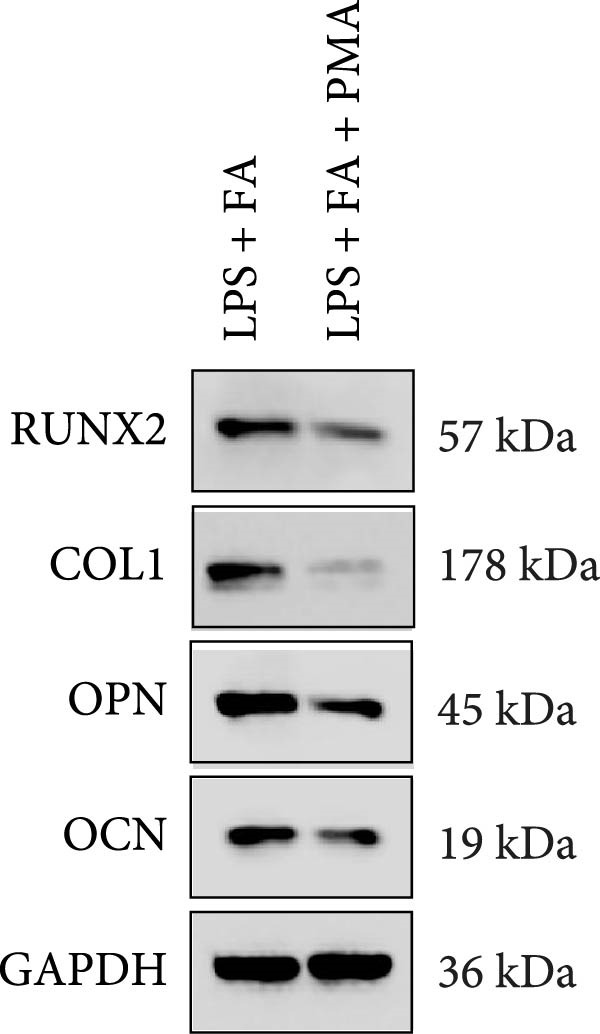
(D)

**Figure:**
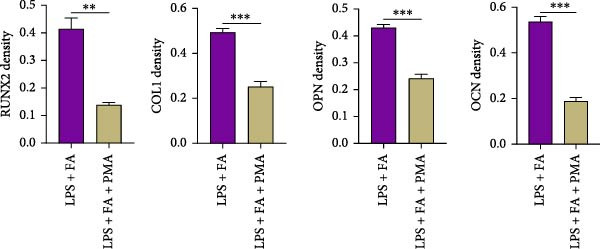
(E)

## 4. Discussion

An inflammatory setting triggered by periodontal microbes drastically diminishes the function of hPDLSCs, leading to alveolar bone destruction and impaired regenerative capacity [34]. Despite the detrimental effects of inflammation on hPDLSC function, their inherent plasticity and pivotal role in tissue regeneration make them an attractive target for bioactive interventions aimed at restoring periodontal health [35, 36]. For instance, Gu et al. demonstrated that diosgenin decreases TNF‐*α*, IL‐1*β*, and IL‐6 levels, while upregulating osteoblast differentiation‐related genes through inhibiting transcription factor NF‐*κ*B level and inflammation response [37]. Crocin enhances osteogenic differentiation of PDLSCs via activating Wnt/*β*‐catenin pathway, as evidenced by upregulated osteogenic markers and mineralization, which is partially inhibited by Wnt inhibitor DKK1 [38], suggesting hPDLSC plasticity is governed by complex signaling components, affecting inflammation and bone formation. This study demonstrated that FA effectively attenuates LPS‐induced inflammatory responses in hPDLSCs, promotes cellular growth and osteogenic differentiation, and reduces apoptosis via inhibiting the NF‐*κ*B pathway, suggesting its therapeutic potential in periodontitis.

Our results showed that LPS stimulation significantly reduced cell viability and increased apoptosis in hPDLSCs, consistent with previous studies indicating that inflammation disrupts the homeostasis of periodontal stem cells [39]. However, FA treatment effectively reversed these effects, suggesting its protective role against LPS‐induced cellular damage. Furthermore, FA enhanced osteogenic differentiation capacity, as evidenced by increased ALP activity and calcium nodule formation, along with upregulated levels of osteogenic markers such as Runx2, COL1, OPN, and OCN protein [38]. Periodontitis reflects a localized immune response within the periodontal environment, immune mediators contribute to periodontal repair, with transforming growth factor beta 1 (TGF‐*β*1) potentially protective [40, 41], and vitamin D linked to better oral outcomes and lower inflammation (CRP) [42], while the inflammatory cytokines like IL‐1*β*, IL‐6, TNF‐*α*, MMP‐8 and granulocyte‐colony stimulating factor (G‐CSF) are involved in alveolar bone loss and connective tissue degradation in periodontitis [43]. In chronic periodontitis, IL‐1*β* and TNF‐*α* were considered to propagate inflammation into deeper connective tissues, promoting loss of attachment, osteoclast activation, and alveolar bone resorption [44]. Adoptive transfer of IL‐10‐producing B cells decreased local IL‐17 and RANKL expression, thereby suppressing osteoclastogenesis and alleviating alveolar bone loss [45]. These findings highlighted that FA suppresses inflammatory responses and facilitates osteogenic processes under inflammatory conditions, indicating its therapeutic potential in periodontitis.

Mechanistically, cytokines are pivotal in dysregulating the bone remodeling balance within the periodontal region. Proinflammatory cytokines, such as TNF‐*α* and IL‐1*β*, stimulate the production of RANKL by osteoblasts, fibroblasts, and lymphocytes [27]. RANKL then activates osteoclastogenesis via the RANKL/RANK signaling axis, thereby promoting bone resorption, a hallmark of periodontitis. This process is naturally counteracted by osteoprotegerin (OPG), a decoy receptor that binds to RANKL and prevents its interaction with RANK, thus inhibiting osteoclast differentiation [46]. The RANKL/OPG ratio is therefore a critical determinant of alveolar bone homeostasis, and its disruption by inflammation is a key driver of periodontal destruction [47]. Our results demonstrate that FA potently inhibits the LPS‐induced phosphorylation of p65 and I*κ*B*α*, the core components of the NF‐*κ*B pathway. By doing so, FA dampens the NF‐*κ*B‐driven transcriptional upsurge of proinflammatory cytokines (TNF‐*α*, IL‐1*β*, and IL‐6), while promoting the secretion of the anti‐inflammatory cytokine IL‐10 [48]. This anti‐inflammatory action is significant because the suppression of TNF‐*α* and IL‐1*β* directly translates to a reduced stimulus for RANKL expression, thereby creating an environment less conducive to osteoclast activation. This aligns with previous findings that FA could inhibit RANKL‐induced osteoclastogenesis and bone erosion in rheumatoid arthritis models by suppressing the NF‐*κ*B pathway [22]. Furthermore, beyond modulating the bone resorptive microenvironment, the inhibition of NF‐*κ*B by FA directly benefits the osteogenic lineage. Chronic NF‐*κ*B activation is known to suppress the expression and activity of key osteogenic transcription factors like Runx2 [49]. The downregulation of NF‐*κ*B by FA thus alleviates this repression, which corroborates our observed upregulation of Runx2, COL1, OPN, and OCN, and the enhanced mineralization. This dual role, inhibiting bone resorption and promoting bone formation, is critically affirmed by our rescue experiment. The addition of PMA, an activator of NF‐*κ*B, significantly reversed the beneficial effects of FA on both inflammation and osteogenesis [26]. This confirms that the anti‐inflammatory and pro‐osteogenic functions of FA are mediated, at least in part, through the specific inhibition of the NF‐*κ*B pathway. Collectively, our study reveals that FA exerts anti‐inflammatory and proregenerative effects on hPDLSCs under LPS‐induced inflammation primarily via the modulation of the NF‐*κ*B pathway, positioning it as a promising multitarget agent for periodontitis therapy.

While the present study primarily elucidates the in vitro mechanistic actions of FA, our findings pave the way for its potential clinical translation as a targeted immunomodulatory and osteo‐promotive agent for periodontitis. The demonstrated efficacy of FA in suppressing inflammation and enhancing osteogenic differentiation of hPDLSCs suggests its promise as a local drug delivery system adjunctive to conventional scaling and root planing. For instance, incorporating FA into hydrogels, nanoparticles, or periodontal chips could allow for sustained release at the inflammatory site, directly modulating the periodontal microenvironment, inhibiting NF‐*κ*B‐driven bone resorption, and promoting the regeneration of alveolar bone [15, 24]. Future studies validating these effects in in vivo animal models and exploring biocompatible delivery platforms will be crucial steps toward developing FA‐based adjunctive therapies for managing progressive periodontitis. Although our results provide strong evidence for the therapeutic potential of FA in periodontitis, several limitations should be acknowledged. First, this study relies on an in vitro model of LPS‐induced single cells. While this model effectively elucidates specific mechanisms, it cannot replicate the complex microenvironment of periodontitis in vivo. Future studies will validate these findings in animal models of periodontitis, which more closely resemble the anatomical and physiological structures of human periodontal tissues. Additionally, more advanced coculture systems can be established, incorporating multispecies microbial biofilms to more realistically replicate the infection process of periodontal pathogens. Second, the FA solution administration method used in this study does not align with the clinical requirement for intraperiodontal pocket delivery, and its local effective concentration, duration of action, and long‐term effects on the oral microecology remain unclear. To address this, the core task in the future is to develop a local sustained‐release formulation of FA and systematically evaluate its drug release kinetics, biodegradability, and ecological impacts on the oral microbiota, thereby providing key data for clinical translation. Finally, this study focused on the osteogenic effect of FA but did not directly evaluate its impact on osteoclast‐mediated bone resorption, failing to fully reveal its regulatory role in the bone metabolic balance of periodontitis. In the future, verification should be conducted in an osteoclast formation system; by observing FA’s effects on RANKL‐induced osteoclast differentiation, bone resorption lacuna formation, and the expression of key markers, its potential for bidirectional regulation of bone metabolism can be comprehensively assessed.

## 5. Conclusion

In conclusion, this study confirms that FA can significantly alleviate LPS‐induced inflammatory responses and promote osteogenic differentiation of hPDLSCs by inhibiting the NF‐*κ*B signaling pathway. We innovatively reveal the direct molecular mechanism underlying FA’s dual anti‐inflammatory and osteogenic effects in the periodontitis microenvironment, and clarify the core role of the NF‐*κ*B pathway in this process. These findings provide solid experimental evidence and a theoretical basis for developing FA into a multifunctional therapeutic strategy targeting both inflammatory control and bone regeneration promotion in periodontitis.

**Nomenclature**
ALP: Alkaline phosphatase
ARS: Alizarin red S
CCK‐8: Cell counting kit‐8
COL1: Collagen type I
FA: Ferulic acid
hPDLSCs: Human periodontal ligament stem cells
IL‐1*β*: Interleukin‐1 beta
IL‐6: Interleukin‐6
IL‐10: Interleukin‐10
LPS: Lipopolysaccharide
OCN: Osteocalcin
OPN: Osteopontin
PMA: Phorbol 12‐myristate 13‐acetate
RANK: Receptor activator of NF‐*κ*B
RANKL: Receptor activator of NF‐*κ*B ligand
Runx2: Runt‐related transcription factor 2
TNF‐*α*: Tumor necrosis factor‐alpha.

## Ethics Statement

Ethical approval was not required for this study because it does not involve any human experiments.

## Consent

The authors have nothing to report.

## Disclosure

All authors read and approved the manuscript.

## Conflicts of Interest

The authors declare no conflicts of interest.

## Author Contributions

All authors contributed to this present work. Qiao Wang and Yongzhi Gao designed the study and drafted the manuscript. Bo Feng acquired and interpreted the data. Qiao Wang revised the manuscript.

## Funding

The study was supported by the Qiqihar Science and Technology Plan Joint Guidance Project (Grant LSFGG‐2025070) with Qiao Wang.

## Supporting Information

Additional supporting information can be found online in the Supporting Information section.

## Supporting information


**Supporting Information** Figure S1. Based on the CCK‐8 assay to evaluate the effects of different concentrations of FA on the viability of hPDLSCs.

## Data Availability

The datasets generated and/or analyzed during the current study are available from the corresponding author, Yongzhi Gao, upon reasonable request.

## References

[1] Papapanou P. N. , Sanz M. , and Buduneli N. , et al.Periodontitis: Consensus Report of Workgroup 2 of the 2017 World Workshop on the Classification of Periodontal and Peri-Implant Diseases and Conditions, Journal of Periodontology. (2018) 89, no. Suppl 1, S173–S182. 29926951 10.1002/JPER.17-0721

[2] Nasrabadi N. , Ramezanian N. , Ghorbanian P. , Forouzanfar A. , and Mohammadipour H. S. , Evaluation of Cytotoxicity and Antimicrobial Activity of Experimental Composites Containing Chitosan-Silver Oxide Particles Against Two Main Pathogenic Bacteria in Periodontal Disease, Protein & Peptide Letters. (2024) 31, no. 2, 97–106, 10.2174/0109298665240242231016103321. 37921156

[3] Hirtz C. , O’Flynn R. , and Voisin P. M. , et al.The Potential Impact of Salivary Peptides in Periodontitis, Critical Reviews in Clinical Laboratory Sciences. (2021) 58, no. 7, 479–492, 10.1080/10408363.2021.1907298. 33849374

[4] Yue M. and Jia R. , Alternative Splicing of Pre-mRNA Matters in Oral Diseases, Current Gene Therapy. (2025) 25, no. 4, 349–359, 10.2174/0115665232302948240718050212. 39075952

[5] He J. , Liu Y. , Xu H. , Wei X. , and Chen M. , Insights Into the Variations in Microbial Community Structure During the Development of Periodontitis and Its Pathogenesis, Clinical Oral Investigations. (2024) 28, no. 12, 10.1007/s00784-024-06074-7. 39617812

[6] Kwon T. , Lamster I. B. , and Levin L. , Current Concepts in the Management of Periodontitis, International Dental Journal. (2021) 71, no. 6, 462–476, 10.1111/idj.12630. 34839889 PMC9275292

[7] Garaicoa-Pazmino C. , Fretwurst T. , and Squarize C. H. , et al.Characterization of Macrophage Polarization in Periodontal Disease, Journal of Clinical Periodontology. (2019) 46, no. 8, 830–839, 10.1111/jcpe.13156, 2-s2.0-85068109199. 31152604

[8] Alghamdi B. , Jeon H. H. , and Ni J. , et al.Osteoimmunology in Periodontitis and Orthodontic Tooth Movement, Current Osteoporosis Reports. (2023) 21, no. 2, 128–146, 10.1007/s11914-023-00774-x. 36862360 PMC10696608

[9] Herrera D. , Sanz M. , and Kebschull M. , et al.Treatment of Stage IV Periodontitis: The EFP S3 Level Clinical Practice Guideline, Journal of Clinical Periodontology. (2022) 49, no. Suppl 24, 4–71. 10.1111/jcpe.13639 35688447

[10] Wang B. , Ge F. , Wang W. , Wang B. , Xian C. J. , and Zhai Y. , Hydrogel-Based Therapeutic Strategies for Periodontal Tissue Regeneration: Advances, Challenges, and Future Perspectives, Pharmaceutics. (2025) 17, no. 11, 10.3390/pharmaceutics17111382, 1382. 41304720 PMC12655002

[11] Qian Y. , Cai B. , and Chi F. , et al.Alveolar Bone Loss and Tooth Loss Contribute to Increase in Cancer Mortality Among Older Patients, BMC Oral Health. (2023) 23, no. 1, 10.1186/s12903-023-03543-5. PMC10731843 38114973

[12] Dong X. and Huang R. , Ferulic Acid: An Extraordinarily Neuroprotective Phenolic Acid With Anti-Depressive Properties, Phytomedicine: International Journal of Phytotherapy and Phytopharmacology. (2022) 105, 10.1016/j.phymed.2022.154355, 154355. 35908520

[13] Antonopoulou I. , Sapountzaki E. , Rova U. , and Christakopoulos P. , Ferulic Acid From Plant Biomass: A Phytochemical With Promising Antiviral Properties, Frontiers in Nutrition. (2021) 8, 10.3389/fnut.2021.777576, 777576. 35198583 PMC8860162

[14] Ou Q. , Zhang S. , and Fu C. , et al.More Natural More Better: Triple Natural Anti-Oxidant Puerarin/Ferulic Acid/Polydopamine Incorporated Hydrogel for Wound Healing, Journal of Nanobiotechnology. (2021) 19, no. 1, 10.1186/s12951-021-00973-7. PMC8359571 34380475

[15] Chatterjee S. and Rajasekar A. , Preparation and Characterization of Ferulic Acid Hydrogel and Its Application as a Local Drug Delivery Agent in Periodontitis, Cureus. (2024) 16, no. 5, 10.7759/cureus.60534, e60534. 38887323 PMC11181101

[16] Bayer J. , Petersen N. K. , Hess J. V. , Jockel-Schneider Y. , and Högger P. , Impact of a Dietary Supplementation With French Maritime Pine Bark Extract Pycnogenol(®) on Salivary and Serum Inflammatory Biomarkers During Non-Surgical Periodontal Therapy-A Randomized Placebo-Controlled Double-Blind Trial, Nutrients. (2025) 17, no. 9, 10.3390/nu17091546, 1546. 40362854 PMC12073762

[17] Chen Y. , Wang H. , and Yang Q. , et al.Single-Cell RNA Landscape of the Osteoimmunology Microenvironment in Periodontitis, Theranostics. (2022) 12, no. 3, 1074–1096, 10.7150/thno.65694. 35154475 PMC8771561

[18] Zhang W. , Jia L. , and Zhao B. , et al.Quercetin Reverses TNF-*α* Induced Osteogenic Damage to Human Periodontal Ligament Stem Cells by Suppressing the NF-*κ*B/NLRP3 Inflammasome Pathway, International Journal of Molecular Medicine. (2021) 47, no. 4, 10.3892/ijmm.2021.4872. PMC7891819 33537804

[19] Jiang J. , Zhang N. , Song H. , Yang Y. , Li J. , and Hu X. , Oridonin Alleviates the Inhibitory Effect of Lipopolysaccharide on the Proliferation and Osteogenic Potential of Periodontal Ligament Stem Cells by Inhibiting Endoplasmic Reticulum Stress and NF-*κ*B/NLRP3 Inflammasome Signaling, BMC Oral Health. (2023) 23, no. 1, 10.1186/s12903-023-02827-0. PMC9999511 36894905

[20] Chen H. , Liu Y. , Yu S. , Li C. , Gao B. , and Zhou X. , Cannabidiol Attenuates Periodontal Inflammation Through Inhibiting TLR4/NF-*κ*B Pathway, Journal of Periodontal Research. (2023) 58, no. 4, 697–707, 10.1111/jre.13118. 37143211

[21] Zhang D. , Jing B. , and Chen Z. N. , et al.Ferulic Acid Alleviates Sciatica by Inhibiting Neuroinflammation and Promoting Nerve Repair via the TLR4/NF-*κ*B Pathway, CNS Neuroscience & Therapeutics. (2023) 29, no. 4, 1000–1011, 10.1111/cns.14060. 36601662 PMC10018085

[22] Doss H. M. , Samarpita S. , Ganesan R. , and Rasool M. , Ferulic Acid, a Dietary Polyphenol Suppresses Osteoclast Differentiation and Bone Erosion via the Inhibition of RANKL Dependent NF-*κ*B Signalling Pathway, Life Sciences. (2018) 207, 284–295, 10.1016/j.lfs.2018.06.013, 2-s2.0-85048723114. 29908722

[23] Liang J. W. , Li P. L. , and Wang Q. , et al.Ferulic Acid Promotes Bone Defect Repair After Radiation by Maintaining the Stemness of Skeletal Stem Cells, Stem Cells Translational Medicine. (2021) 10, no. 8, 1217–1231, 10.1002/sctm.20-0536. 33750031 PMC8284777

[24] Du K. , Li Z. , Fang X. , Cao T. , and Xu Y. , Ferulic Acid Promotes Osteogenesis of Bone Marrow-Derived Mesenchymal Stem Cells by Inhibiting microRNA-340 to Induce *β*-Catenin Expression Through Hypoxia, European Journal of Cell Biology. (2017) 96, no. 6, 496–503, 10.1016/j.ejcb.2017.07.002, 2-s2.0-85026463413. 28764862

[25] Dong S. , Jia L. , and Sun S. , et al.TAZ Reverses the Inhibitory Effects of LPS on the Osteogenic Differentiation of Human Periodontal Ligament Stem Cells Through the NF-*κ*B Signaling Pathway, BMC Oral Health. (2024) 24, no. 1, 10.1186/s12903-024-04497-y. PMC11210133 38926705

[26] Chen M. , Lin X. , Zhang L. , and Hu X. , Effects of Nuclear Factor-*κ*B Signaling Pathway on Periodontal Ligament Stem Cells under Lipopolysaccharide-Induced Inflammation, Bioengineered. (2022) 13, no. 3, 7951–7961, 10.1080/21655979.2022.2051690. 35297308 PMC9208442

[27] Ahern E. , Smyth M. J. , Dougall W. C. , and Teng M. W. L. , Roles of the RANKL–RANK Axis in Antitumour Immunity—Implications for Therapy, Nature Reviews Clinical Oncology. (2018) 15, no. 11, 676–693, 10.1038/s41571-018-0095-y, 2-s2.0-85053785067. 30232468

[28] Wang J. and Zhu Y. , FOXA1 Knockdown Alleviates Inflammation and Enhances Osteogenic Differentiation of Periodontal Ligament Stem Cells via STAT3 Pathway, Journal of Orthopaedic Surgery and Research. (2024) 19, no. 1, 10.1186/s13018-024-05286-7. PMC11613586 39623394

[29] Baladehi R.-F. , Memar M.-Y. , and Sales A.-J. , et al.The Effect of Oncogene Proteins of Human Papillomaviruses on Apoptosis Pathways in Prostate Cancer, Oncologie. (2022) 24, no. 2, 227–245.

[30] Fatima S. , Song Y. , and Zhang Z. , et al.Exploring the Pharmacological Mechanisms of P-Hydroxylcinnamaldehyde for Treating Gastric Cancer: A Pharmacological Perspective With Experimental Confirmation, Current Molecular Pharmacology. (2024) 17, 10.2174/0118761429322420241112051105, e18761429322420. 39660529

[31] Xiong G. , Ji W. , and Wang F. , et al.Quercetin Inhibits Inflammatory Response Induced by LPS From *Porphyromonas gingivalis* in Human Gingival Fibroblasts via Suppressing NF-*κ*B Signaling Pathway, BioMed Research International. (2019) 2019, 10, 10.1155/2019/6282635, 2-s2.0-85072042287, 6282635. 31531360 PMC6720363

[32] Hepokur C. , Misir S. , Cicek M. , Habtemariam S. , and Sharifi-Rad J. , Protective Effect of Salvia Cadmica on Fibroblast Cells From t-Bhp-Induced Oxidative Damage, Current Pharmaceutical Analysis. (2024) 20, no. 3, 178–187, 10.2174/0115734129293569240327093703.

[33] Chen D. , Xu L. , and Xing H. , et al.Sangerbox 2: Enhanced Functionalities and Update for a Comprehensive Clinical Bioinformatics Data Analysis Platform, IMeta. (2024) 3, no. 5, 10.1002/imt2.238, e238. 39429873 PMC11487553

[34] Wen S. , Zheng X. , and Yin W. , et al.Dental Stem Cell Dynamics in Periodontal Ligament Regeneration: From Mechanism to Application, Stem Cell Research & Therapy. (2024) 15, no. 1, 10.1186/s13287-024-04003-9. PMC11526537 39482701

[35] Ern C. , Berger T. , Frasheri I. , Heym R. , Hickel R. , and Folwaczny M. , Differentiation of hMSC and hPDLSC Induced by PGE2 or BMP-7 in 3D Models, Prostaglandins, Leukotrienes, and Essential Fatty Acids. (2017) 122, 30–37, 10.1016/j.plefa.2017.06.005, 2-s2.0-85021736026. 28735626

[36] Zhao Z. , Sun Y. , and Qiao Q. , et al.Human Periodontal Ligament Stem Cell and Umbilical Vein Endothelial Cell Co-culture to Prevascularize Scaffolds for Angiogenic and Osteogenic Tissue Engineering, International Journal of Molecular Sciences. (2021) 22, no. 22, 10.3390/ijms222212363, 12363. 34830243 PMC8621970

[37] Cong S. , Peng Q. , and Cao L. , et al.Diosgenin Prevents Periodontitis by Inhibiting Inflammation and Promoting Osteogenic Differentiation, Oral Diseases. (2024) 30, no. 4, 2497–2510, 10.1111/odi.14708. 37593795

[38] Gu Y. and Bai Y. , Osteogenic Effect of Crocin in Human Periodontal Ligament Stem Cells via Wnt/*β*-Catenin Signaling, Oral Diseases. (2024) 30, no. 3, 1429–1438, 10.1111/odi.14523. 36705490

[39] Yu H. , Wang P. , and Lu H. , et al.Effects of G-CSF on hPDLSC Proliferation and Osteogenic Differentiation in the LPS-Induced Inflammatory Microenvironment, BMC Oral Health. (2023) 23, no. 1, 10.1186/s12903-023-03040-9. PMC10294445 37365568

[40] Yang Y. , Liu W. , Wei J. , Cui Y. , Zhang D. , and Xie J. , Transforming Growth Factor-*β*1-Induced N-Cadherin Drives Cell-Cell Communication Through Connexin43 in Osteoblast Lineage, International Journal of Oral Science. (2021) 13, no. 1, 10.1038/s41368-021-00119-3. PMC8044142 33850101

[41] Rowaiye A.-B. , Njoku M.-O. , and Oli A.-N. , et al.In Vivo Effects of Aqueous Extract of *Gongronema latifolium* Benth on the Tumor Necrosis Factor-*α*, Transforming Growth Factor-*β* , And Hepatic Enzymes. (2021) 23, no. 4, 547–557.

[42] Isola G. , Alibrandi A. , Rapisarda E. , Matarese G. , Williams R. C. , and Leonardi R. , Association of Vitamin D in Patients with Periodontitis: A Cross-Sectional Study, Journal of Periodontal Research. (2020) 55, no. 5, 602–612, 10.1111/jre.12746. 32173876

[43] Akram Z. , Abduljabbar T. , Sauro S. , and Daood U. , Effect of Photodynamic Therapy and Laser Alone as Adjunct to Scaling and Root Planing on Gingival Crevicular Fluid Inflammatory Proteins in Periodontal Disease: A Systematic Review, Photodiagnosis and Photodynamic Therapy. (2016) 16, 142–153, 10.1016/j.pdpdt.2016.09.004, 2-s2.0-84992126230. 27619532

[44] Graves D. T. and Cochran D. , The Contribution of Interleukin-1 and Tumor Necrosis Factor to Periodontal Tissue Destruction, Journal of Periodontology. (2003) 74, no. 3, 391–401, 10.1902/jop.2003.74.3.391, 2-s2.0-0038505147. 12710761

[45] Shi T. , Jin Y. , Miao Y. , Wang Y. , Zhou Y. , and Lin X. , IL-10 Secreting B Cells Regulate Periodontal Immune Response during Periodontitis, Odontology. (2020) 108, no. 3, 350–357, 10.1007/s10266-019-00470-2. 31701299

[46] Braz-Silva P. H. , Bergamini M. L. , Mardegan A. P. , De Rosa C. S. , Hasseus B. , and Jonasson P. , Inflammatory Profile of Chronic Apical Periodontitis: A Literature Review, Acta Odontologica Scandinavica. (2019) 77, no. 3, 173–180, 10.1080/00016357.2018.1521005, 2-s2.0-85059091941. 30585523

[47] Fadli N. A. , Abdul Rahman M. , Karsani S. A. , and Ramli R. , Oral and Gingival Crevicular Fluid Biomarkers for Jawbone Turnover Diseases: A Scoping Review, Diagnostics. (2024) 14, no. 19, 10.3390/diagnostics14192184, 2184. 39410587 PMC11475764

[48] Cao L. , Li Z. , and Yang Z. , et al.Ferulic Acid Positively Modulates the Inflammatory Response to Septic Liver Injury Through the GSK-3*β*/NF-*κ*B/CREB Pathway, Life Sciences. (2021) 277, 10.1016/j.lfs.2021.119584, 119584. 33961853

[49] Chen J. , Yu M. , Li X. , Sun Q. F. , Yang C. Z. , and Yang P. S. , Progranulin Promotes Osteogenic Differentiation of Human Periodontal Ligament Stem Cells via Tumor Necrosis Factor Receptors to Inhibit TNF-*α* Sensitized NF-kB and Activate ERK/JNK Signaling, Journal of Periodontal Research. (2020) 55, no. 3, 363–373, 10.1111/jre.12720. 31854464

