# Supplementary figures and images for: Ferulic Acid Alleviates Inflammation and Promotes Osteogenic Differentiation in Periodontitis by Inhibiting NF‐κB Pathway
Source: Stem Cells Int. 2025 Dec 17;2025:1891956. doi: 10.1155/sci/1891956 (PMC12767440; doi:10.1155/sci/1891956)

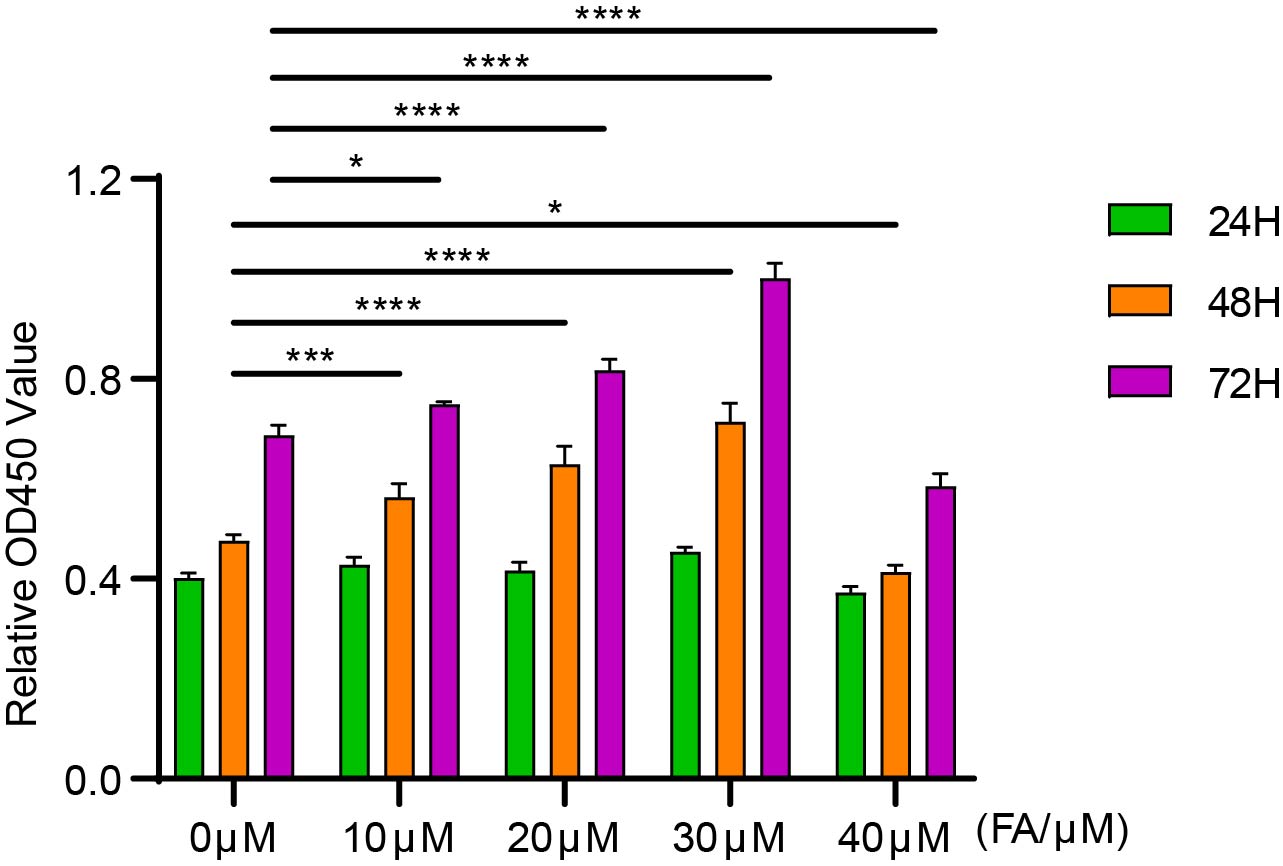

Supplement: Supplementary file 1 — Supporting Information Figure S1. Based on the CCK‐8 assay to evaluate the effects of different concentrations of FA on the viability of hPDLSCs. [file SCI-2025-1891956-s001.jpg]
